# Patient-centred care, health behaviours and cardiovascular risk factor levels in people with recently diagnosed type 2 diabetes: 5-year follow-up of the ADDITION-Plus trial cohort

**DOI:** 10.1136/bmjopen-2015-008931

**Published:** 2016-01-06

**Authors:** Hajira Dambha-Miller, Andrew J M Cooper, Rebecca K Simmons, Ann Louise Kinmonth, Simon J Griffin

**Affiliations:** 1 Primary Care Unit, Department of Public Health and Primary Care, University of Cambridge, Cambridge, UK; 2 MRC Epidemiology Unit, University of Cambridge School of Clinical Medicine, Institute of Metabolic Science, Cambridge Biomedical Campus, Cambridge, UK

**Keywords:** Type 2 diabetes, PRIMARY CARE, patient-centred care

## Abstract

**Objective:**

To examine the association between the experience of patient-centred care (PCC), health behaviours and cardiovascular disease (CVD) risk factor levels among people with type 2 diabetes.

**Design:**

Population-based prospective cohort study.

**Setting:**

34 general practices in East Anglia, UK, delivering organised diabetes care.

**Participants:**

478 patients recently diagnosed with type 2 diabetes aged between 40 and 69 years enrolled in the ADDITION-Plus trial.

**Main outcome measures:**

Self-reported and objectively measured health behaviours (diet, physical activity, smoking status), CVD risk factor levels (blood pressure, lipid levels, glycated haemoglobin, body mass index, waist circumference) and modelled 10-year CVD risk.

**Results:**

Better experiences of PCC early in the course of living with diabetes were not associated with meaningful differences in self-reported physical activity levels including total activity energy expenditure (β-coefficient: 0.080 MET h/day (95% CI 0.017 to 0.143; p=0.01)), moderate-to-vigorous physical activity (β-coefficient: 5.328 min/day (95% CI 0.796 to 9.859; p=0.01)) and reduced sedentary time (β-coefficient: −1.633 min/day (95% CI −2.897 to −0.368; p=0.01)). PCC was not associated with clinically meaningful differences in levels of high-density lipoprotein cholesterol (β-coefficient: 0.002 mmol/L (95% CI 0.001 to 0.004; p=0.03)), systolic blood pressure (β-coefficient: −0.561 mm Hg (95% CI −0.653 to −0.468; p=0.01)) or diastolic blood pressure (β-coefficient: −0.565 mm Hg (95% CI −0.654 to −0.476; p=0.01)). Over an extended follow-up of 5 years, we observed no clear evidence that PCC was associated with self-reported, clinical or biochemical outcomes, except for waist circumference (β-coefficient: 0.085 cm (95% CI 0.015 to 0.155; p=0.02)).

**Conclusions:**

We found little evidence that experience of PCC early in the course of diabetes was associated with clinically important changes in health-related behaviours or CVD risk factors.

**Trial registration number:**

ISRCTN99175498; Post-results.

**Strengths and limitations of this study**

- Our study is the first to use objective measures of health behaviours to examine the impact of patient-centred care in recently diagnosed type 2 diabetes.
- The study duration is 5 years with high rates of follow-up.
- We included a large number of general practitioner (GP) surgeries that reflects the average UK GP list size, number of doctors/nurses and diabetes prevalence.
- Patient-centred care was only measured at a single time point.
- The majority of our participants were Caucasian males with high levels of education and employment, thereby limiting the generalisability of our findings.

## Introduction

Type 2 diabetes is a common condition mostly managed in general practice. Despite current lifestyle and medication treatments, patients with diabetes still have high rates of cardiovascular disease (CVD) morbidity and mortality.^1^ Patient-centred care (PCC) is considered the cornerstone of UK general practice and may play an important role in the management of CVD risk factor levels.^2^ By understanding individual health beliefs, considering patient preferences and developing mutual management plans, general practitioners (GPs) may be able to positively influence health behaviours such as diet, physical activity, smoking and alcohol intake, each of which are known to influence CVD risk factor levels.^3^ This potentially effective and cost-effective role for GPs in influencing patient health behaviours has recently been emphasised in national and international health policy.^4^ ^5^ ^6^ The majority of supporting evidence comes from observational data reporting inverse associations between PCC and CVD risk factor levels.^7^ Trial findings have been more variable with some studies reporting no effect from interventions promoting PCC, while others report reduced CVD risk factor levels including glycated haemoglobin (HbA1c), blood pressure, cholesterol and body mass index (BMI).^3^ ^8^ ^9^ ^10^ There is currently insufficient evidence to confirm whether PCC influences CVD risk factor levels among patients with diabetes, and the mechanism to explain any associations remains unclear. We hypothesise that the mechanism linking PCC to CVD risk factor levels is through patient health behaviours.^11^

The majority of diabetes care occurs in general practice where there is increasing pressure on GP consultation time. This is leading to a range of alternative chronic disease management strategies such as more routinised care, telehome care and remote monitoring, each of which may diminish PCC.^12^ ^13^ ^14^ With the need to optimise efficiency as well as effectiveness in diabetes care, it is increasingly important to assess the experience of PCC in improving disease risk. Evidence for the role of PCC in cost-effective diabetes care is needed to inform policy and has implications for the management of chronic disease more widely.

We aimed to quantify the association between the experience of PCC delivered by GPs and CVD risk factor levels at 1-year and 5-year follow-up in a well-characterised cohort of patients recently diagnosed with type 2 diabetes. To enable better understanding of the potential mechanisms underlying this association, we also examined associations between PCC and health behaviours.

## Methods

### Study design

A detailed description of the *ADDITION-Plus* study design and rationale can be found elsewhere.^15^ In brief, *ADDITION-Plus* is a randomised-controlled trial among 34 general practices across East Anglia, UK. *ADDITION-Plus* examined the efficacy of a facilitator-led, theory-based behaviour change intervention for individuals with recently diagnosed type 2 diabetes. In total, 478 out of 1109 eligible individuals agreed to participate and were individually randomised to receive either intensive treatment alone (n=239) or intensive treatment plus a facilitator-led individual behaviour change intervention (n=239). The trial was not designed to influence patient–practitioner interactions and there were no differences in PCC measures, health behaviours, CVD risk factor levels between trial groups at 1 year, and no differences in the proceeding multivariate analyses between trial arms. Therefore, data for this analysis were pooled and treated as a cohort analysis. Participants in the trial were followed up for 5 years. All measurements were taken at baseline, 1-year and 5-year follow-up, except for objectively measured physical activity which was assessed at 1-year and 5-year follow-up, and PCC at 1-year follow-up. All participants gave written informed consent, and the study was approved by the Eastern Multi-Centre Research Ethics Committee (reference number 02/5/54). The trial is registered as ISRCTN99175498.

### Measurements and outcomes

#### Self-reported health behaviour

Physical activity and dietary intake were assessed by self-report using the validated EPIC Physical Activity Questionnaire (EPAQ-2) and semiquantitative food frequency questionnaire.^16^ ^17^ Alcohol intake and smoking status (categorised as never smoked, ex-smoker or current smoker) were assessed by self-report questionnaire.

#### Objective measures of health behaviour

Physical activity was measured using a combined heart rate and movement sensor (Actiheart, CamNtech) worn for at least three consecutive days, as described previously.^16^ Resulting time-series data were summarised into physical activity energy expenditure (in kJ/kg/day)—a measure of total physical activity, sedentary time (h/day) and moderate-to-vigorous physical activity (MVPA; min/day).^18^ Plasma vitamin C levels (which offer an objective biomarker measure of fruit and vegetable intake)^17^ were measured using a Fluoroskan Ascent FL fluorometer.^17^ ^19^

#### Clinical and biochemical measures

Clinical and biochemical measures were collected by trained staff following standardised protocols, as described elsewhere.^15^ Blood pressure was calculated as the mean of three measurements using an automatic sphygmomanometer. Body weight and height were measured in light clothing and without shoes using a scale (SECA) and a fixed rigid stadiometer, respectively.^15^ Venous blood samples were collected for analysis of lipid levels and HbA1c. Modelled 10-year cardiovascular risk was calculated using the UK Prospective Diabetes Study (UKPDS) risk engine (V.3.0).

#### Patient-centred care

PCC is a challenging concept to study or measure as there are multiple definitions and tools within the literature. At its core, PCC seeks to encompass the management of biophysical markers, alongside the human experience of disease. The consultation and relational empathy (CARE) measure is a holistic tool that attempts to capture PCC with a focus on the quality of consultations in terms of the ‘human’ aspects (empathic process of care). This is in the context of a doctor–patient interaction and from the patient’s perspective. The CARE questionnaire is a measure that has been shown to be meaningful to patients, acceptable and easy to complete. It has been developed and extensively validated within the primary care setting, where the vast majority of type 2 diabetes care occurs in the UK.^20^ The CARE measure includes 10 questions based on a Likert scale ranging from 1 to 5. A CARE summary measure was derived by summing the individual scores from the 10 individual questions, with a possible range of 10–50.^20^ Participants completed a questionnaire on PCC experiences in relation to their GPs over the preceding year, in relation to diabetes care using the CARE measure at 1-year follow-up.^21^

### Statistical analysis

Participant characteristics were summarised at baseline, 1-year and 5-year follow-up using means (SDs) or frequencies. Participants with incomplete data across time points were excluded from the analyses. Multivariate linear regression models were constructed to examine the prospective associations between baseline and 1-year follow-up, and between 1-year and 5-year follow-up between PCC measures and: (1) change in self-reported health behaviours; (2) change in objective health behaviours; (3) changes in biochemical and clinical measures; and (4) change in modelled 10-year cardiovascular risk. As physical activity was not measured objectively at baseline, this was examined cross-sectionally at 1 year. All models were adjusted, based on a priori reasoning, for age, sex, socioeconomic group, ethnicity, trial group, relevant medication use (ie, change in blood pressure, lipid or diabetes medications). Statistical analysis was performed using STATA/SE V.13.1 (STATA-Corp, College Station, Texas, USA). Statistical significance was set at p<0.05.

## Results

### Participant characteristics

Three hundred and ninety-six *ADDITION-Plus* participants had complete data and were included in these analyses. Participants had a mean (SD) age of 61 (6.9) years; the majority were Caucasian (96%) and male (63%; table 1). Seventy-four per cent of participants were in part-time or full-time employment and most continued in full-time education after the age of 16 years (61%). Baseline mean (SD) HbA1c was 7.1 (1.4)% (49.7 mmol/mol) (1.3). Change in clinical and biochemical variables at baseline, 1-year and 5-year follow-up is summarised in table 2. Mean BMI, waist circumference, HbA1c, blood pressure and cholesterol levels improved over the 5 years of follow-up. The mean (SD) CARE score was 39 (9.8) at 1-year follow-up. There were no significant differences in age, sex, ethnicity, employment status, social class, education, smoking status, blood pressure, lipid profile, waist circumference and 10-year modelled cardiovascular risk between participants with and without missing data for these analyses.

**Table 1:** Baseline characteristics in the ADDITION-Plus trial cohort (n=396)

Variables	Mean±SD
*Sociodemographic characteristics*	
Male sex, n (%)	252 (63.1)
Age at baseline (years)	61 (6.9)
White ethnic origin, n (%)	379 (96)
Employed, n (%)	296 (74)
Social class, n (%)	
High	170 (43.4)
Manual	173 (44.3)
Non-manual	48 (12.2)
Education, n (%)	
Full-time education finished at <16 years	150 (38.4)
Full-time education finished at 16–18 years	172 (44.0)
Full-time education finished at >18 years	69 (17.6)
*Medical history*	
History of angina, n (%)	47 (10.7)
History of hypertension, n (%)	175 (40.2)
History of any cardiovascular disease including AF	50 (11.6)
History of myocardial infarct, n (%)	31 (7.26)
History of hypercholesterolaemia, n (%)	197 (46.9)
History of stroke, n (%)	13 (3.1)
*Self-reported drug use*	
Any glucose-lowering drug, n (%)	126 (32)
Any antihypertensive drug, n (%)	280 (71)
Any cholesterol-lowering drug, n (%)	205 (52)
*Self-reported lifestyle*	
Physical activity energy expenditure, mean (SD) (kJ/kg/day)	29 (7.4)
Smoking status, n (%)	
Current smoker	55 (14)
Ex-smoker	196 (49.7)
Never smoker	142 (36)
Alcohol per week (units), mean (SD)	9 (13.9)

Values are presented as mean (SD) unless specified.

**Table 2:** Clinical variables of participants with complete data at all three time points

	N	Baseline	One-year follow-up	Five-year follow-up
Clinical characteristics				
BMI (kg/m²)	383	32.4 (5.6)	31.8 (5.1)	31.9 (5.4)
Waist circumference (cm)	383	109.9 (13.0)	108.6 (12.8)	107.9 (13.6)
HbA1c (%), mean (SD)	387	7.1 (1.4)	6.6 (0.9)	6.9 (0.9)
Systolic blood pressure (mm Hg)	396	136.8 (19.7)	130.2(17.7)	132.2 (16.4)
Diastolic blood pressure (mm Hg)	396	80.7 (10.6)	76.4 (9.5)	73.9 (9.8)
Total cholesterol (mmol/L)	390	4.9 (1.06)	4.3 (0.8)	4.2 (0.9)
HDL-cholesterol (mmol/L)	390	1.2 (0.3)	1.2 (0.3)	1.3 (0.3)
LDL-cholesterol (mmol/L)	390	2.9 (0.9)	2.3 (0.7)	2.1 (0.7)

Values are mean (SD).

BMI, body mass index; HbA1c, glycated haemoglobin; HDL, high-density lipoprotein; LDL, low-density lipoprotein.

### Self-reported health behaviours

Analysis of change from baseline to 1-year follow-up showed that participants reporting better experiences of PCC were more likely to increase their self-reported physical activity by small amounts, including total activity energy expenditure (β-coefficient: 0.080 MET h/day (95% CI 0.017 to 0.143), MVPA (β-coefficient: 5.328 min/day (95% CI 0.796 to 9.859)), and reduce sedentary time (β-coefficient: −1.633 min/day (95% CI −2.897 to −0.368); table 3). We observed no clear associations between PCC and self-reported diet or alcohol intake. Over a longer follow-up from 1 to 5 years, there was no clear evidence that better experiences of PCC were associated with change in self-reported physical activity, diet or alcohol intake (table 3). We have not reported on change in smoking status as too few (n=12) participants quit or started smoking to enable this to be examined.

**Table 3:** Linear associations between patient-centred care and outcomes at 1- and 5-year follow-up in ADDITION-Plus cohort

		Changes from 0 to 1 years of follow-up				Changes from 1 to 5 years of follow-up			
Variable	N	Coefficient		95% CI	p Value	Coefficient		95% CI	p Value
Self-reported measures									
Total activity energy expenditure (MET h/day)	371	0.080	0.017	0.143	0.01	−0.037	−0.318	0.243	0.79
Sedentary time (min/day)	371	−1.633	−2.897	−0.368	0.01	0.014	−0.010	0.037	0.25
Moderate-to-vigorous physical activity (min/day)	371	5.328	0.796	9.859	0.01	−0.241	−0.880	0.400	0.46
Energy intake (kJ/day)	371	0.920	−3.960	5.810	0.71	0.012	0.001	0.001	0.51
Alcohol per week (units)	371	0.022	−0.037	0.081	0.47	−0.022	−0.085	0.041	0.49
Objectively measured health behaviours									
Physical activity energy expenditure (kJ/kg/day)*	308	−0.001	−0.166	0.164	0.99	−0.014	0.850	−0.100	0.08
Plasma vitamin C (µmol/L)	303	−0.231	−0.462	<0.001	0.05	−0.040	−0.100	0.020	0.17
Clinical and biochemical measures									
HbA1c (%)†	387	−0.006	−0.015	0.004	0.23	0.004	−0.005	0.013	0.39
Systolic blood pressure (mm Hg)‡	396	−0.561	−0.653	−0.468	0.01	0.107	−0.053	0.267	0.19
Diastolic blood pressure (mm Hg)‡	396	−0.565	−0.654	−0.476	0.01	0.064	−0.031	0.159	0.19
Total cholesterol (mmol/L)§	390	0.002	−0.006	0.011	0.58	0.001	−0.008	0.010	0.83
HDL-cholesterol (mmol/L)§	390	0.002	0.001	0.004	0.03	−0.002	−0.004	0.001	0.17
LDL-cholesterol (mmol/L)§	390	0.007	0.001	0.014	0.07	0.001	−0.007	0.007	0.99
Waist circumference (cm)	383	−0.060	−0.120	0.011	0.07	0.085	0.015	0.155	0.02
BMI (kg/m²)	383	−0.010	−0.031	0.006	0.19	0.013	−0.010	0.036	0.27
Modelled UKPDS 10-year cardiovascular risk¶	390	0.001	−0.001	<0.001	0.54	0.001	−0.001	0.001	0.51

*Measured at 1-year only.

†Adjusted for sex, age, ethnicity, social class and hypoglycaemic medication.

‡Adjusted for sex, age, ethnicity, social class and antihypertensive medication.

§Adjusted for sex, age, ethnicity, social class and lipid-lowering therapy.

¶Adjusted for sex, age, ethnicity, social class, lipid-lowering therapy, antihypertensive and hypoglycaemic medication.

BMI, body mass index; HbA1c, glycated haemoglobin; HDL, high-density lipoprotein; LDL, low-density lipoprotein; UKPDS, UK Prospective Diabetes Study.

### Objective health behaviours

Over the first year of follow-up, there was no evidence that better experience of PCC were associated with objectively measured physical activity or diet (fruit and vegetable intake measured with plasma vitamin C levels). Similarly, analysis of change between 1 and 5 years also demonstrated no associations between PCC and objectively measured diet or physical activity. These results are summarised in table 3.

### Clinical and biochemical measures

Analysis of change over the first year of follow-up demonstrated that participants with better experiences of PCC had marginally greater increases in high-density lipoprotein (HDL) cholesterol (β-coefficient: 0.002 mmol/L (95% CI 0.001 to 0.004)) and decreases in both systolic blood pressure (β-coefficient: −0.561 mm Hg (95% CI −0.653 to −0.468)) and diastolic blood pressure (β-coefficient: −0.565 mm Hg (95% CI −0.654 to −0.476)). As shown in table 3, there were no other associations between baseline and 1 year in clinical and biochemical measures. Over the longer 5-year follow-up, there were no associations between PCC and clinical or biochemical outcomes, except for waist circumference (β-coefficient: 0.085 cm (95% CI 0.015 to 0.155)) which increased with higher PCC.

## Discussion

Better experience of PCC early after the diagnosis of type 2 diabetes was associated with a small, but not clinically meaningful change in self-reported physical activity, time spent sedentary, and improvements in HDL-cholesterol and blood pressure at 1-year. This was not reflected in the objective measures of physical activity. Over the longer term, we found no evidence to suggest that PCC was associated with changes in health behaviours or CVD risk factor levels. This study provides insufficient evidence that patients recently diagnosed with type 2 diabetes who have experiences of PCC are more likely to have lower cardiovascular risk factor levels via changes in patient health behaviours.

To the best of our knowledge, this is the first study to use objective measures of health behaviours alongside self-reported health behaviours to quantify the impact of experiences of PCC in a population with recently diagnosed type 2 diabetes. Furthermore, it includes a relatively long duration of follow-up of 5 years. We observed discrepancies in associations between PCC and self-reported, and objectively measured physical activity and diet. This highlights the potential bias associated with patient self-report questionnaires in previous studies. Other strengths include the use of a large number of GP surgeries which reflect average UK GP list sizes, diabetes prevalence, doctor or nurse whole time equivalent and patient experiences of diabetes care. Further, more than 50% of practices that were approached agreed to participate in the original study. The participant follow-up rate was also high at 95% at year follow-up and 83% at the 5-year follow-up. In relation to previous literature on PCC, this cohort study also includes a relatively large sample size. Additional strengths include our measure of PCC; while some previous studies have used non-specific and non-validated patient satisfaction questionnaires as a marker of PCC, we used the validated CARE measure.^8^ ^10^ ^21^ ^22^ The validity and reliability of the CARE measure has been extensively demonstrated, and applied in over 3000 general practice consultations in areas of high and low deprivation and across multiple health conditions.

A number of limitations of our study also warrant discussion. We measured PCC at a single time point at 1-year follow-up which may explain differences between 1-year and 5-year results. Further, because doctor–patient relationships are dynamic and are established or changed over time,^23^ we were not able to examine how changes in experiences of PCC might affect health behaviours and CVD risk factor levels. The majority of participants were Caucasian males with high levels of education and employment, thereby limiting the generalisability of our findings as experiences of PCC and diabetes care may differ in a more ethnically diverse or socially deprived populations. The majority of participants reported high CARE scores which, due to homogeneity, will likely have reduced our ability to identify associations with health outcomes. Finally, we also conducted a number of hypothesis tests and as a result we cannot exclude the role of chance as a plausible explanation for our findings.

Previous studies examining the association of interventions to alter PCC and CVD risk factor levels in type 2 diabetes have reported mixed results. This may be related to the fact that PCC is a broad term with multiple descriptions and measures, and therefore a high level of heterogeneity exists between studies on this subject.^24^ We found positive associations, albeit clinically not meaningful, between PCC and self-reported physical activity level, blood pressure and HDL-cholesterol, that is—people with better PCC experiences reported being more physically active and had higher HDL-cholesterol levels and lower blood pressures at 1-year. This is consistent with some previous observational and trial data,^22^ ^25^ ^26^ ^27^ ^28^ except our study includes objective measures and therefore overcomes some of the limitations associated with previous self-reported data. Several studies have also reported inverse associations between PCC and non HDL-cholesterol,^25^ ^28^ ^22^ BMI, HbA1c^29^ ^30^ ^31^ and cardiovascular risk.^32^ We did not observe such associations at 1-year or 5-year follow-up.^10^ ^25^ ^30^ Differences may have been because our study was underpowered to detect these changes, or might be related to our measure of PCC. Our study is the first to use the CARE measure as a specific marker of PCC focusing on empathy in patients recently diagnosed with type 2 diabetes that were followed up over a 5-year period.^20^ These differences, as well as the potential role of chance, may also explain the positive, albeit small, unexpected association between PCC and waist circumference.

Further, baseline measures also vary across studies which may explain differences in findings. For example, mean HbA1c in participants in our cohort at baseline was 7.1%. A recent large study in type 2 diabetes within secondary care demonstrated significant reductions in HbA1c following a PCC intervention.^31^ This study suggested that a PCC approach may be most effective in improving glycaemic control when baseline HbA1c is over 8.5%, and reported modest effects in patients with an HbA1c below 7%. Previous studies in primary care have similarly demonstrated a greater effect of PCC when baseline HbA1c was high.^22^ ^29^ ^30^ ^33^ We therefore carried out a post hoc analysis including only participants with HbA1c over 8.5% at baseline, and found stronger associations between PCC, physical activity, and HbA1c, non-HDL-cholesterol and BMI, but these associations did not reach statistical significance, likely owing to the reduction is sample size and therefore statistical power.

The literature is bedeviled with lack of clear definition and measures of PCC in terms of interactions with health professionals.^24^ ^34^ ^35^ More frequent use of standardised and validated measures of PCC in future research will reduce heterogeneity and allow comparison between studies on PCC. Further, most studies use self-reported measures of health behaviours which are prone to reporting error and bias, as demonstrated by the lack of consistency between our subjective and objective assessments. Social desirability bias may be one explanation for the higher levels of self-reported health behaviours compared with objective health behaviours observed in our study. This highlights the need for future research to include objective measures of outcomes. Further, we could not exclude reverse causality as a potential explanation for this and previous findings. Future well-conducted trials alongside qualitative work are essential to explore the mechanism linking PCC, health behaviours and outcomes. Also, we found stronger associations between PCC among people with poor glycaemic control, albeit not significant. This has been suggested previously,^31^ and future research will need to stratify disease severity and patient groups to further examine the role of PCC in these particular groups of patients.

Current National Health Service (NHS) healthcare policy emphasises the importance of ‘making every contact count’, and highlights the role that GPs have to play in modifying health behaviours and secondary disease risk. Our study provides insufficient evidence to exclude that PCC is associated with improvements in health-related behaviours or CVD risk factor levels in the first 5 years following diagnosis. Although PCC is preferred by our patients and often considered a moral imperative or the ‘right thing’ for clinicians to do, it is important to adequately balance PCC against evidence-based disease management strategies in type 2 diabetes.^36^
